# Harnessing the highly adaptable barnase-barstar system for genetic biocontrol of *Aedes aegypti*

**DOI:** 10.1038/s42003-025-08588-6

**Published:** 2025-08-04

**Authors:** Katherine Nevard, Joshua X. D. Ang, Michelle A. E. Anderson, Estela Gonzalez, Lewis Shackleford, Luke Alphey

**Affiliations:** 1 Arthropod Genetics, The Pirbright Institute, Pirbright, UK; 2 The Department of Biology, University of York, York, UK; 3 York Biomedical Research Institute, University of York, Heslington, UK; 4 Present Address: Animal and Plant Health Agency, Woodham Lane, Surrey, UK

**Keywords:** Synthetic biology, Genetic engineering

## Abstract

Toxin-antidote pairs can be used in gene drive systems, providing powerful means to modify mosquito populations. Here we use the toxin-antidote pair, barnase and barstar, originally identified in *Bacillus amyloliquefaciens*, due to their high binding affinity, small size and lack of need for cofactors. In *Ae. aegypti* cell culture, we find that barnase can kill and barstar can rescue the effect. Ubiquitous expression of barnase in transgenic mosquitoes results in up to 100% lethality. Tissue specific expression results in flightless or reduced fertility in females and this could be partially rescued by ubiquitous expression of barstar likely due to insufficient expression of barstar in affected tissues. In conclusion, we show barnase-barstar to be a highly adaptable toxin-antidote pair, providing a basis for developing toxin-antidote gene drive systems.

## Abstract (web summary)

The barnase-barstar pair is effective in *Aedes aegypti* as a basis for toxin-antidote gene drive systems, barnase is able to kill mosquitoes and cells and barstar rescues the effect in a ubiquitous or tissue-specific manner.

## Introduction

A*edes aegypti*, the mosquito vector of dengue, Zika, yellow fever, and chikungunya viruses, is a continuing problem in the world today, causing diseases in millions of people every year^1–4^. With climate change expanding the geographical range of *Ae. aegypti*, and consequently the viruses it transmits^5,6^, along with the rise of insecticide resistance in this species^7–9^, novel control methods are urgently needed. Sterile Insect Technique, *Wolbachia*- and homing-based genetic biocontrol methods have made remarkable progress in *Ae. aegypti* within the past decade and are potential candidates for the control of *Aedes* mosquitoes^10–17^. However, the utility of each method depends highly on the preference of the communities where the modified mosquitoes could be released. This necessitates the development of a broad range of alternative strategies that communities and programme managers can select based on their specific requirements. While the aforementioned technologies can be modified to provide a wide range of persistence and/or invasiveness, an alternative class of local gene drive systems, which allow for the spread of a favourable genetic trait that can be highly persistent but also non-invasive, could be more desirable.

One example is the underdominance system. Underdominance, also known as negative heterosis, classically refers to situations where hybrids of two pure-breeding strains exhibit lower fitness than either parental type. This tends to result in frequency-dependent fitness effects, so that in mixed populations the rarer type is at a relative disadvantage as these will typically mate the more common type, producing lower-fitness hybrids. Designs for synthetic underdominance systems can be comprised of two paired toxin-antidote effectors^18–20^. Conceptually, this involves two distinct genetic elements, each carrying a toxin and a complementary antidote: one construct encodes toxin A paired with antidote B, while the second encodes toxin B paired with antidote A. Viability is thus maintained only in individuals possessing both genetic elements. Such systems, introduced into a wild-type population, exhibit an unstable equilibrium—at allele frequencies above this equilibrium the frequency of transgenic individuals tends to increase, as mating between transgene homozygotes and wild-type (WT) individuals leads to toxins and antidotes segregating, resulting in descendants with minimal or zero fitness, whereas mating among transgene homozygotes produces healthy offspring. Below the unstable equilibrium, the transgenes tend to decrease in frequency. Consequently, the wild-type population is gradually transformed into a modified population expressing a desired trait, such as refractoriness to pathogens, encoded alongside the transgene. Following establishment, a stable boundary between modified and unmodified populations emerges, as rare wild-type migrants into the transformed population suffer the frequency-dependent disadvantage characteristic of underdominance systems—and similarly for rare transgenic migrants into adjacent wild-type populations. This characteristic is particularly attractive from economic and regulatory perspectives, since once established, the transgenics persist for an extended period, yet remain spatially contained and non-invasive, minimising spread into neighboring populations. Another toxin-antidote system, Killer-Rescue, requires only a single toxin-antidote pair^21,22^. Killer-Rescue has different dynamics, being temporally and spatially limited; this system may be very useful for intermediate steps or limited field trials before full deployment.

Despite its promising characteristics, the development of synthetic underdominance systems in *Ae. aegypti* and other insect species have been hampered by the lack of a broadly applicable and effective toxin-antidote pair. Here, we explore the possibility of utilising in *Ae. aegypti*, the barnase-barstar (Bn-Bs) system, originally found in the bacterium *Bacillus amyloliquefaciens*, in particular for genetic biocontrol applications. In *Bacillus amyloliquefaciens*, Bn (110 amino acids) acts as an extracellular ribonuclease while Bs (89 amino acids) is a specific inhibitor that binds to Bn, neutralising the otherwise lethal effect of the ribonuclease activity ^23^. Due to the potent ribonuclease activity of Bn and the ability of Bs to repress it, the pair has been used to enforce outcrossing (heterosis) in a range of plants^24–27^. Here we show that the pair works similarly in *Ae. aegypti* cell culture (Aag2) and subsequently demonstrate that Bs can rescue the lethality of Bn in transgenic *Ae. aegypti*, as long as the expression of Bs overlaps with that of Bn. This adds a highly adaptable effector to the arsenal of genetic biocontrol of *Ae. aegypti* and potentially other insects.

## Results

### Bn is lethal in Aag2 cells and can be rescued with Bs

**Fig. 1:**
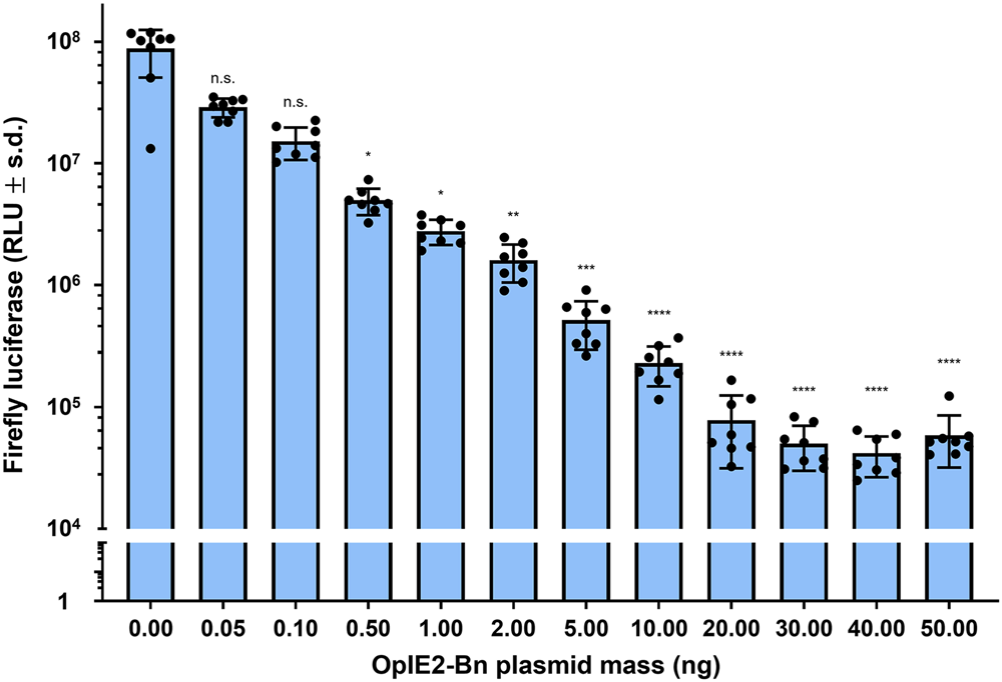
Transfection of *OpIE2*-Bn causes lethality in Aag2 cells. Transfected cell viability is measured as a function of relative light units (RLU) produced by the luciferase assay. Mean values are indicated by the tip of each bar plot. Error bars are standard deviations. Statistical significance was estimated using Kruskal–Wallis test, followed by Dunn’s multiple comparison test relative to the RLU of cells treated with 0 ng of OpIE2-Bn plasmid (adjusted *p* values: *p* > 0.05 ^ns^, *p* < 0.01^**^, *p* < 0.001^***^, *p* < 0.0001^****^).

**Fig. 2:**
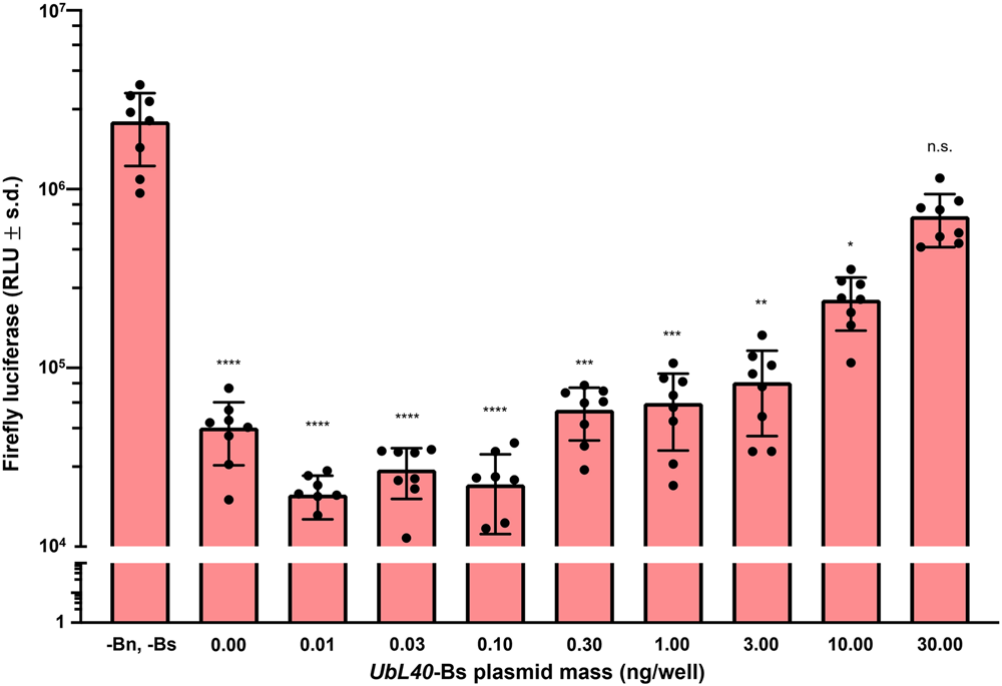
*UbL40*-Bs rescues lethality caused by co-transfection of 10 ng/well of *OpIE2*-Bn. Mean values are indicated by the tip of each bar plot. Error bars are standard deviations. Error bars are standard deviations. Statistical significance was estimated using Kruskal–Wallis test, followed by Dunn’s multiple comparison test relative to the RLU of cells treated with neither *OpIE2*-Bn nor *UbL40*-Bs plasmid (adjusted *p* values: *p* > 0.05 ^ns^, *p* < 0.05^*^, *p* < 0.01^**^, *p* < 0.001^***^, *p *< 0.0001^****^).

The killing effect of Bn was first assessed in vitro by transfecting Aag2 cells with a plasmid expressing *PUb*-Firefly luciferase (*PUb*-Fluc) as a proxy for cell viability and varying concentrations of a separate plasmid expressing Bn from the *OpIE2* promoter. Fluc activity was found to decrease with increasing concentrations of Bn. It is conceivable that Bn, a ribonuclease, is specifically degrading Fluc RNA, but its broad specificity—cuts 5’ of G residues—and known ability to induce cell death in other systems, suggest that Bn induces cell death in the transfected cells (Fig. 1). Next, Aag2 cells were transfected with *PUb*-Fluc, 10 ng/well of *OpIE2*-Bn, and varying concentrations of a plasmid expressing Bs from the *UbL40* promoter. Fluc activity was shown to gradually increase with increasing concentrations of *UbL40*-Bs compared to cells with neither *OpIE2*-Bn nor *UbL40*-Bs, indicating that the expression of Bs reverses the killing effect of Bn in Aag2 cells (Fig. 2).

### Bn expression in vivo can affect a range of tissues in *Ae. aegypti*

**Fig. 3:**
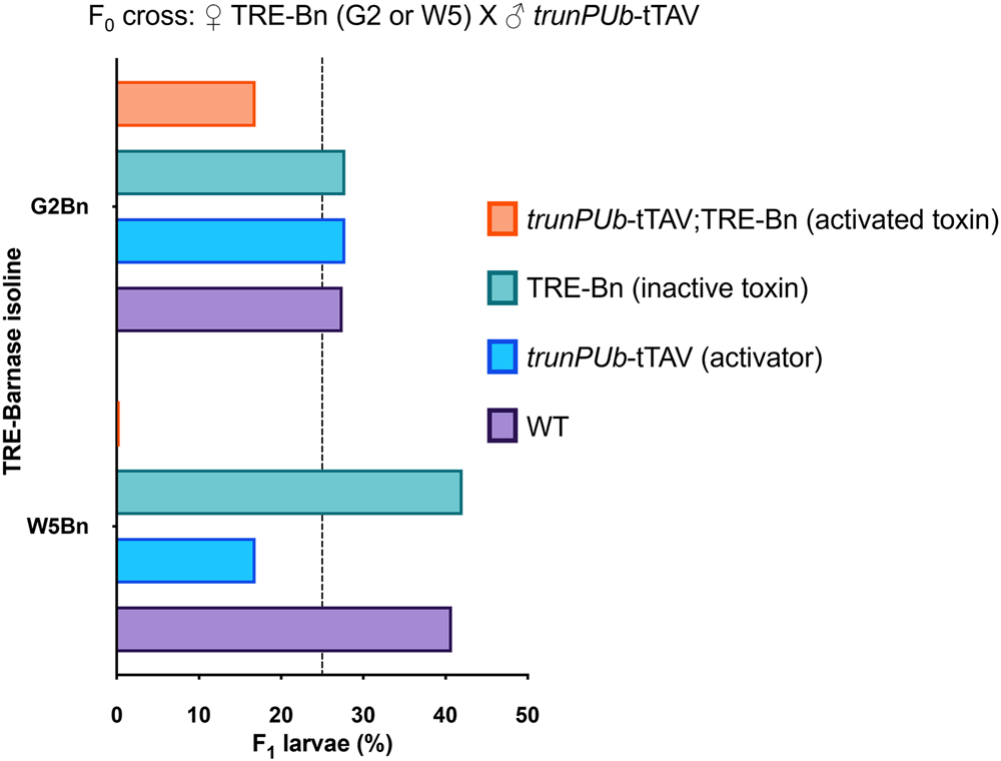
TRE-Bn activated by *trunPUb*-tTAV causes lethality in *Ae. aegypti.* Black dashed line indicates the expected proportion (25%) of each genotype if lethality is not observed. Note that the *trunPUb* promoter in *trunPUb*-tTAV has a reduced intron in the 5’UTR compared to the *PUb* promoter in *PUb*-Bs previously shown to reduce expression^28^.

Of the eight *Ae*. *aegypti* transgenic isolines expressing *PUb*-Bs-TRE-Bn established, two (G2 and W5) were selected for the assessments below based on genotype and sex ratio, indicating that both carried single transgene insertions and were not linked to the M/m loci associated with sex determination (Supplementary Tables 1 and 2). These transgenics express Bs controlled by the *polyubiquitin* promoter (*PUb*-Bs) and Bn under the control of a tetracycline-responsive element (TRE-Bn). Bs is expressed autonomously, but Bn expression is only activated in the presence of a synthetic tetracycline-repressible transactivator (tTAV). After successfully separating the two elements using Cre (Supplementary Fig. 1), we crossed the two TRE-Bn isolines (hereafter named G2Bn and W5Bn to distinguish from the G2 and W5 isolines initially generated) to transgenic lines expressing tTAV ubiquitously (*trunPUb*-tTAV)^28^. Under Mendelian inheritance, each of the four potential progeny genotypes *trunPUb*-tTAV/TRE-Bn, *trunPUb*-tTAV, TRE-Bn, and non-transgenic should be present at equal frequency, i.e., 25%. However, upon screening the progeny, only 16.9% (*χ* ^2^, df.3, *n* = 295, *p* = 0.02) of larvae from isoline G2 carried both *trunPUb*-tTAV and TRE-Bn, while no such double heterozygotes (*χ* ^2^, df.3, *n* = 485, *p* < 0.0001) were found for isoline W5Bn, indicating that the *trunPUb*-tTAV-induced expression of TRE-Bn is fully lethal in the W5Bn isoline (Fig. 3). A decrease in viability in progeny inheriting only *trunPUb*-tTAV was also observed in crosses with W5Bn, but not with G2Bn (discussed further below). Since complete lethality was observed for isoline W5Bn, further experiments were carried out with this isoline.

**Fig. 4:**
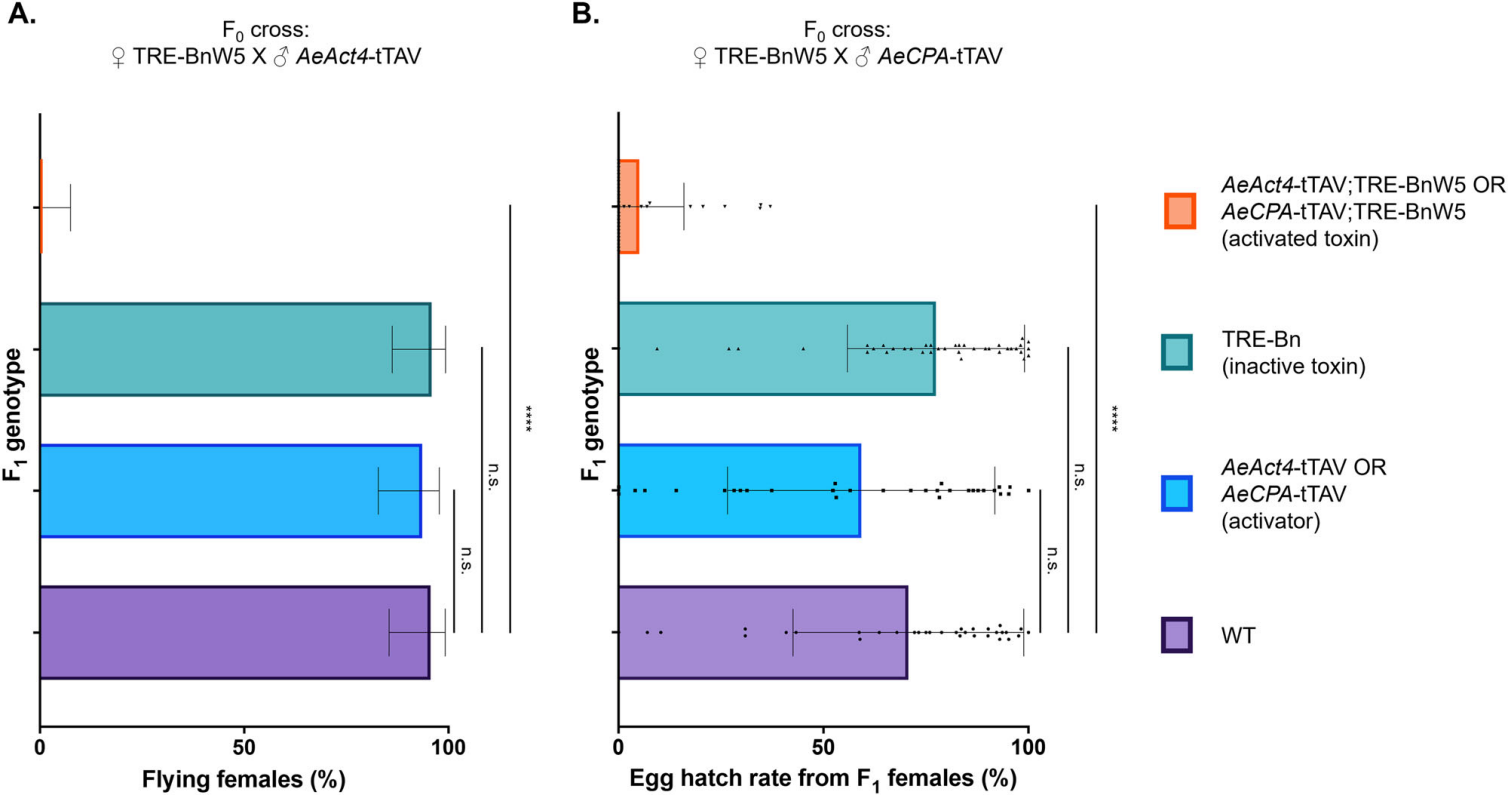
Bn expression in various tissues negatively impacts mosquitoes. **A** Percentage of females that were visibly flying in a cage 3–4 days post eclosion. Error bars are the Wilson 95% confidence intervals for the binomial proportion. Statistical significance was estimated using Fisher’s exact test (two-tailed) relative to the proportion of flying WT females (*p* > 0.05 ^ns^, *p* < 0.0001^****^). **B** Percentage of eggs that successfully hatched 5 days after laying. Mean values are indicated by the tip of each bar plot. Error bars are standard deviations. Individual data points correspond to the hatch rate from each female. Statistical significance was estimated using Kruskal–Wallis test followed by Dunn’s multiple comparison relative to the egg hatch rates from WT females (*p* > 0.05 ^ns^, *p* < 0.0001^****^).

We then crossed the W5Bn isoline to transgenic mosquitoes expressing tTAV in female indirect flight muscle (*AeAct4*-tTAV). As expected, targeting muscles essential for female flight resulted in a dramatic reduction in flight ability (Fisher’s exact test, *n* = 93, *p* < 0.0001) with no female *AeAct4*-tTAV/TRE-Bn transheterozygotes being able to fly (Fig. 4A, Supplementary Table 3). We also crossed the W5Bn isoline to transgenics expressing tTAV in the female midgut post bloodfeeding (*AeCPA*-tTAV). Female progeny with the four resulting genotypes were crossed to WT males and assessed for the number of eggs laid and the egg hatch rate. We did not find any statistically significant differences (Kruskal–Wallis test, *n* = 89, *p* > 0.67) in the number of eggs laid between WT and the three other genotypes (Supplementary Table 4) but egg hatch rates were reduced from an average of 70.7% in WT to 5.1% (Kruskal–Wallis test, *n* = 71, *p* < 0.0001) in transheterozygous females expressing Bn in the midgut (Fig. 4B, Supplementary Table 4).

In the progeny of both crosses, genotype frequencies (Supplementary Table 3) did not deviate significantly from Mendelian expectations at the L4 larval stage (*AeAct4*-tTAV: *χ* ^2^, df.3, *n* = 538, *p* = 0.06; *AeCPA*-tTAV: *χ* ^2^, df.3, *n* = 1066, *p* = 0.81). Collectively, these findings demonstrate that tissue-specific fitness effects can be achieved through the use of tissue-specific regulatory elements to drive Bn expression.

### Bs rescues mosquitoes from the effects of Bn

**Fig. 5:**
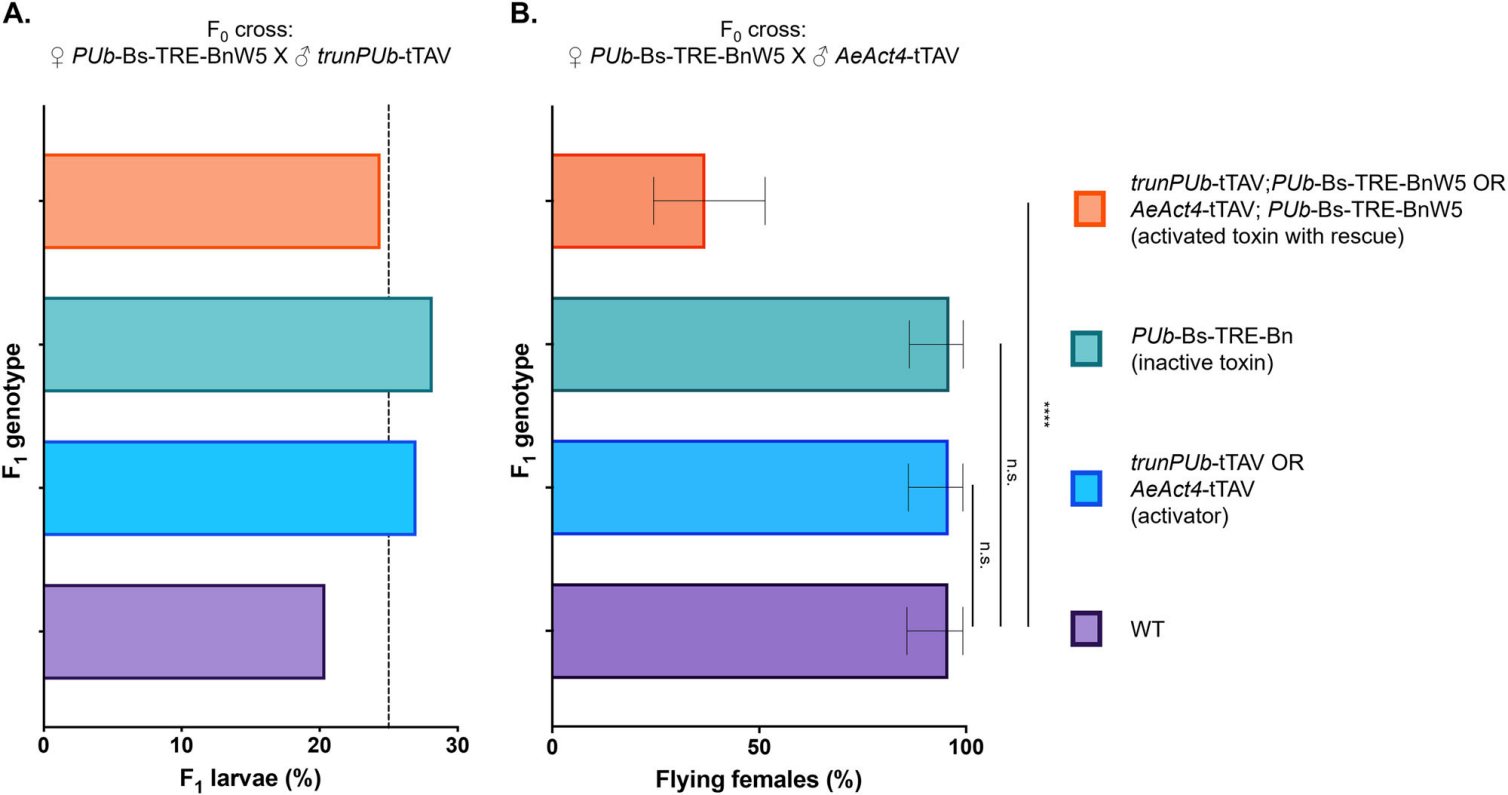
Bs can rescue mosquitoes from the deleterious effects of Bn. **A** Percentage of viable larvae of different genotypes (*n* = 348). Black dashed line indicates the expected proportion (25%) of each genotype if lethality is not observed. **B** Percentage (*n* = 50) of female adults that were visibly flying in a cage 3–4 days post eclosion. Error bars are the Wilson 95% confidence intervals for the binomial proportion. Statistical significance was estimated using Fisher’s exact test (two-tailed) relative to the proportion of flying WT females (*p* > 0.05 ^ns^, *p* < 0.0001^****^).

To assess the rescue ability of Bs, the initial *PUb*-Bs-TRE-BnW5 isoline was crossed to the *trunPUb*-tTAV line. The number of viable transheterozygous larvae screened from this cross was not significantly different from their non-transgenic siblings (χ^2^, df.3, *n* = 348, *p* = 0.18), demonstrating the rescue effect of Bs (Fig. 5A, Supplementary Table 5). Notably, progeny inheriting *trunPUb*-tTAV in this cross did not exhibit the reduced viability observed in Fig. 3A. We do not have any clear explanation for the apparent difference; however, this relates to a genotype with neither Bn nor Bs transgenes. High expression levels of tTAV are known to be deleterious^29^, however, this is unlikely to be the explanation here, as the *trunPUb* promoter used to drive tTAV expression was selected for its moderate, ubiquitous expression, shown in cell culture to have lower expression than the full-length promoter fragment^28^. Supporting this, no fitness effects were observed in Fig. 5A. In any case, while the tet-off system is very useful for testing components, as its binary nature and conditional (tetracycline-repressible) expression each facilitate the assembly of genetic systems comprising lethal genes, it is not necessary for applications such as Killer-Rescue or underdominance-based gene drives, in which the antidote element–here barstar–neutralises the lethal effect. If this configuration is ultimately employed as a component of an underdominance system, it will be essential to carefully evaluate the fitness costs associated with each transgene to enable accurate modelling of the system’s effectiveness.

A partial rescue effect was observed in transheterozygotes for *PUb*-Bs-TRE-BnW5/*AeAct4*-tTAV; with 17/46 eclosed females (37%) observed to fly normally (Fig. 5B, Supplementary Table 6). Despite an improvement in flight ability, the transheterozygotes were still significantly worse than their WT siblings (Fisher’s exact test, *n* = 93, *p* < 0.0001), possibly due to expression of Bn and Bs from different promoters. Bs may not be expressed in female indirect flight muscles at a level high enough to fully rescue flight in all individuals. Since we observed incomplete rescue of *AeAct4*-Bn with *PUb*-Bs, we did not assess the latter’s ability to rescue the hatch rate of eggs produced by *PUb*-Bs-TRE-BnW5/*AeCPA*-tTAV.

## Discussion

We have shown the Bn-Bs system from *B*. *amyloliquefaciens* to be functional in *Ae. aegypti*, to our knowledge, the first use in insects to date. We found that the inducible expression of Bn can cause lethality in mosquitoes and that the simultaneous expression of Bs can rescue this effect. *PUb*-Bs did not fully rescue the flightless phenotype caused by Bn expressed in indirect flight muscles (*AeAct4*-tTAV;TRE-BnW5), suggesting that the level of expression from *PUb*^30^ was insufficient in the indirect flight muscles, not surprising since *AeAct4*^29^ is highly expressed in this tissue. We did, however, see a complete rescue of the lethal phenotype with *PUb*-Bs and *trunPUb*-tTAV;TRE-BnW5 expression. The *trunPUb* promoter has reduced expression compared to the full *PUb* promoter of *PUb*-Bs, so we would expect stronger, overlapping expression of Bs, which may be important for complete suppression. In order to translate this system into another organism, care should be taken in promoter selection, as for complete rescue, it is vital that Bs expression overlaps Bn in terms of expression pattern. Furthermore, our cell culture data further suggests that Bs expression levels should be greater than Bn. Coupling its effectiveness as a toxin-antidote with the very short coding sequences of both components, this system represents a use of this toxin-antidote pair that could easily be adapted for genetic control methods in mosquitoes, and potentially other insect species.

These results indicate that the pair could provide the basis for toxin/antidote-based gene drive systems. While Killer-Rescue requires a single toxin-antidote pair, most synthetic underdominance-based designs require two orthogonal pairs. However, this does not necessarily require two distinct toxins—and corresponding antidotes. The expansion of transgenesis in mosquitoes in recent years has led to the characterisation of an increased number and range of regulatory elements^13–16,31^. This could allow the development of an underdominance system using just one toxin-antidote pair if two distinct promoters with non-overlapping expression patterns are used. For example, one construct would have promoter A driving toxin expression and promoter B driving antidote expression, while the other construct would have promoter B driving toxin expression and promoter A driving antidote expression. As long as these two promoters do not overlap in their expression patterns, this arrangement could successfully create the conditions required for underdominance. Alternatively, Bn-Bs (as toxin-antidote A) could be combined with a second toxin-antidote system, such as CRISPR/Cas9 targeting an essential gene (toxin B) and its rescue sequence (antidote B)^20^, to form a functional underdominance system.

## Methods

### Plasmids and cloning

Plasmids AGG1829 (*OpIE2*-Bn)^32^, AGG1984 (*UbL40*-Bs-SV40)^33,34^, AGG1983 (*UbL40*-mCherry-SV40) were synthesised by Twist Bioscience. To generate AGG1767 (*PUb*-Bs-TRE-Bn), the *PUb*-Bs and Bn fragments were synthesised by Twist Bioscience. First *PUb*-Bs was cloned into the DraIII and AsiSI sites of AGG1546^35^. Then the Bn fragment was cloned into the SpeI site of AGG1546+*PUb*-Bs. Complete plasmid sequences are available in NCBI: AGG1829 (PQ260750), AGG1894 (PQ260751), AGG1983 (PQ260747), AGG1767 (PQ260749), AGG1029 *AeCPA*-tTAV (PQ260745), AGG1506 *AeAct4*-tTAV (PQ260746), AGG1522 *trunPub*-tTAV (PQ260748).

### Assessing Bn lethality in cells

Aag2 (*Ae aegypti*) cells were seeded at 5 × 10^4^ cells/well in a 96-well plate. After 24 h the cells were transfected with 50 ng/well AGG1747 (*PUb*-Firefly luciferase)^33^, increasing concentrations of AGG1829 (*OpIE2*-Bn), and a DsRed expressing plasmid to maintain an equal total plasmid mass (100 ng) per well. Eight replicate wells were transfected using Transit-Pro (Mirius) for each condition according to the manufacturer’s instructions. Forty-eight hours later, the cells were washed once with phosphate-buffered saline and lysed in 50 µl 1× passive lysis buffer (Promega). A luciferase assay was performed with the Luciferase Assay Reagent II (Promega), using 3 µl of lysate and read on a GloMax according to the manufacturer’s instructions^30^.

### Assessing the rescue ability of Bs in cells

Aag2 (*Ae aegypti*) cells (kind gift of R. Fragkoudis) were seeded at 5 × 10^4^ cells/well in a 96-well plate. After 24 h, the cells were transfected with 50 ng/well AGG1747 (*PUb*-Firefly luciferase), 10 ng/well AGG1829 (*OpIE2*-Bn), increasing concentrations of AGG1984 (*UbL40*-Bs) and an mCherry expressing plasmid (AGG1983) to maintain an equal total plasmid mass (100 ng) per well. Eight replicate wells were transfected using Transit-Pro (Mirius) for each condition according to the manufacturer’s instructions. Forty-eight hours later, the cells were harvested in 1× passive lysis buffer (Promega), and a luciferase assay was performed with the Luciferase Assay Reagent II (Promega) and read on a GloMax Explorer as described above^30^.

### Mosquito rearing

All experiments performed for this study were approved by the Biological Agents and Genetic Modification Safety Committee of The Pirbright Institute and undertaken in accordance with all relevant ethical regulations. Mosquitoes were reared at 28 +/−1 °C, 60–70% relative humidity on a 12/12 day-night/light cycle with 1 hour of dawn and dusk. Cages of adult mosquitoes were provided with 10% sucrose solution *ad libitum,* and females were bloodfed with defibrinated horse blood (TCS Bioscience) through a Hemotek membrane feeding system (Hemotek Ltd), with a parafilm (Merck) membrane. Females were allowed to lay eggs onto wet filter paper, and eggs were hatched in a vacuum chamber to degas the water and synchronise hatching. Early instar larvae were fed Liquifry (Interpet) and subsequently ground Tetramin flakes (Tetra). Liverpool^13^ served as the WT strain for crosses and microinjection.

### Generation of transgenic lines

WT females were allowed to lay eggs in the dark for 35 min. Less than 1 h post-oviposition, eggs were collected, aligned on damp filter paper, transferred to double-sided tape (3 M) on a plastic cover-slip and injected as Jasinkiene et al.^36^. Injection mix comprised 500 ng/${{{\rm{\mu }}}}$L of donor plasmid AGG1767 (*PUb*-Bs-TRE-Bn) and 300 ng/${{{\rm{\mu }}}}$L of helper plasmid AGG1245 (*PUb*-hyperactive piggyBac transposase)^13^. Five days post-injection, G_0_ eggs were hatched in a vacuum chamber and reared as above. Adult G_0_ males were put into cages in groups of approximately 10 and crossed with WT females in a 1:5 ratio. Females were put into cages in groups of approximately 10 and crossed with WT males in a 1:1 ratio. Each cage was deemed a separate pool. Two days after crossing, the cages were bloodfed, and G_1_ eggs were collected from each pool. Eggs were hatched, and late larvae were screened for the presence of fluorescent markers. Transgenic larvae were reared to adulthood and crossed individually to WT to establish separate isolines. Males were crossed with WT females in a 1:5 ratio, and females with WT males in a 1:1 ratio. A total of 87 transgenic mosquitoes were generated from 8/12 pools of G₀ survivors (Supplementary File 1).

To establish separate *PUb*-Bs and TRE-Bn lines, male *PUb*-Bs-TRE-Bn from the established isolines were crossed to transgenic females expressing *shu*-Cre^35^. Males transheterozygous for *PUb*-Bs-TRE-Bn;*shu*-Cre were then crossed to WT, and the progeny were screened for separation of the fluorescent markers (Supplementary Table 1). Individuals that possessed only *PUb*-Bs (mCherry) or TRE-Bn (ZsYellow) markers were pool crossed to WT to establish separate *PUb*-Bs and TRE-Bn lines.

To activate the transcription of the Bn in the isolines above, we used previously validated ubiquitous (*trunPUb*-tTAV, AGG1522), female indirect flight muscle-specific (*AeAct4*-tTAV, AGG1506), and female midgut-specific (*AeCPA*-tTAV, AGG1029) tTAV-expressing transgenic isolines to activate Bn expression (P. Leftwich, personal communication).

### Flight testing

50 female pupae of each genotype were placed into cages and allowed to eclose. Three to four days post eclosion, flight ability was assessed through observation. Flight was encouraged by tapping the area of the cage where the mosquito rested, as Navarro et al.^37^. Flying adults were removed from the cage, and non-flyers were reassessed for flight ability using the same method one to two days later to confirm their status.

### Fertility testing

Approximately four days post eclosion, 50 females of each of the four genotypes were crossed in cages with 25 WT males. Six days later, females were allowed to blood feed for four hours, and non-bloodfed females were removed from the cage one day later. Three days after blood feeding, females were transferred into modified 24-well tissue culture plates containing 2% agarose (EAgaL plates), and the fertility assay was carried out as Tsujimoto and Adelman^38^, but without the use of image analysis software. Females were allowed to lay eggs in the plates for ~24 h, then were returned to the cage so the assay could be repeated through the second oviposition. Eggs were counted under a dissecting microscope, then vacuum hatched five days after they were laid. One to two days after hatching, L1 larvae were chilled on ice to immobilise them and were counted under a dissecting microscope.

### Statistics and reproducibility

The exact number of mosquitoes and repetitions for each experiment are indicated in the Supplementary Data File 1, Figures and/or the Figure legends. Data analysis and graph production were done using GraphPad Prism 10.

For comparisons between control and multiple treatment groups involving continuous, non-normally distributed data (Figs. 1, 2, and 4B), statistical significance was assessed using the Kruskal–Wallis test followed by Dunn’s post-hoc multiple comparison test. To control the false discovery rate, the Benjamini, Krieger, and Yekutieli two-stage linear step-up procedure was applied to the resulting *p* values.

For categorical data (Figs. 3, 4A, and 5), statistical significance between control and treatment groups was evaluated using Fisher’s exact test (two-tailed). Chi-square tests were employed to determine significant deviations from expected Mendelian inheritance ratios.

### Reporting summary

Further information on research design is available in the Nature Portfolio Reporting Summary linked to this article.

## Supplementary information

Supplementary Information

Supplementary Data 1

Description of Additional Supplementary Files

Reporting Summary

## Data Availability

All data generated for this study are available in the main manuscript and supplementary files. Full plasmid sequences are publicly available from NCBI accession numbers: PQ260745-PQ260751. Plasmids are available through Addgene numbers: 242438-242441.
