# Supplementary material for: Harnessing the highly adaptable barnase-barstar system for genetic biocontrol of Aedes aegypti
Source: Commun Biol. 2025 Aug 4;8:1154. doi: 10.1038/s42003-025-08588-6 (PMC12322067; doi:10.1038/s42003-025-08588-6)
Supplement: Supplementary file 1 — Supplementary Information [file 42003_2025_8588_MOESM1_ESM.pdf]

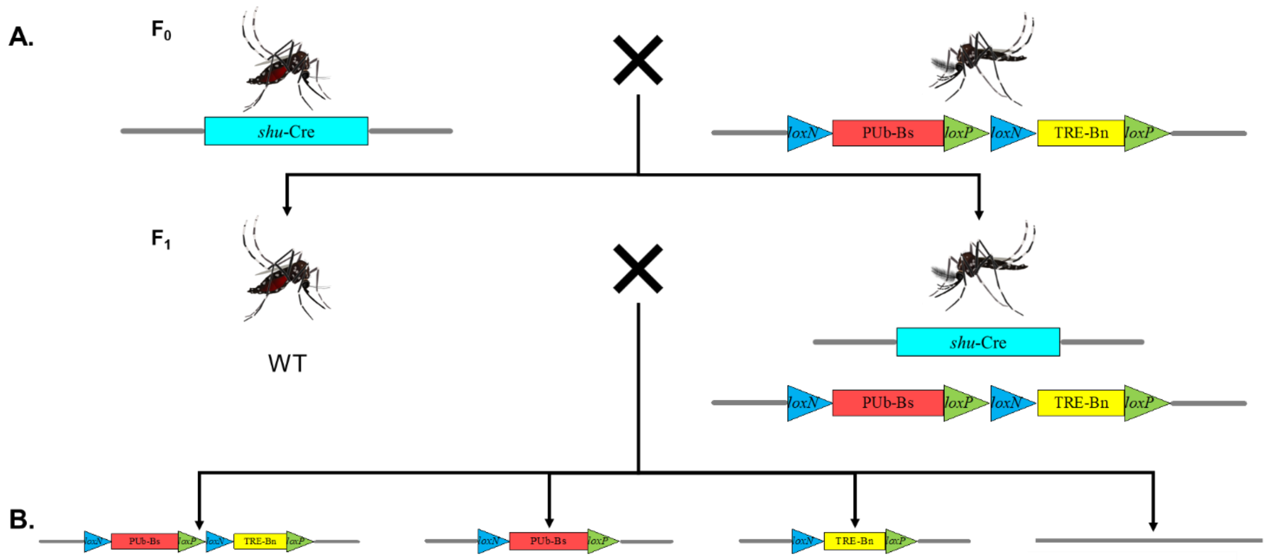

F_1 cross	F_2 progeny (%)			
	PUb-Bs-TRE-Bn	*PUb*-Bs	TRE-Bn	WT
WT X *shu*-Cre;*PUb*-Bs-TRE-BnG2	167 (40.8)	24 (5.9)	20 (4.9)	198 (48.4)
WT X *shu*-Cre;*PUb*-Bs-TRE-BnW5	428 (49.0)	10 (1.1)	7 (0.8)	429 (49.1)

Supplementary Figure 1. Cre recombinase mediates recombination of the TRE-Bn/*PUb*-Bs transgene. (A) Female mosquitoes expressing shu-Cre were crossed to males expressing TRE-Bn/*PUb*-Bs in the F_0 to generate F_1 males carrying both transgenes. (B) Cre-mediated recombination of the TRE-Bn/*PUb*-Bs transgene occurs in the male germline, resulting in progeny carrying only either *PUb*-Bs or TRE-Bn. Mosquito figures are obtained from Ramirez[1,2].

Supplementary Table 1. Transgene integration rates.

Construct ID	Donor template	Number of G0s	Number of pools	Number of positive pools	Number of positive G1s	Total screened	Integration rate (%)
AGG1767	Plasmid	64	12	8	87	613	12.50

Supplementary Table 2. Generation 2 screening results.

Larvae/pupae							Pupae					
Line	Gen 1 Cross (female: male)	+	%	-	%	Total	M	%	F	%	Total	Comment
C2	LVP:1767C2	107	47	120	53	227	0	0	100	100	100	m linked
D3	LVP:1767D3	52	77	16	24	68	20	43	27	57	47	multiple insertions
E	LVP:1767E	77	64	43	36	120	34	51	33	49	67	multiple insertions
G2	LVP:1767G2	141	47	160	53	301	59	59	41	41	100	
N1	1767N1:LVP	43	49	45	51	88	25	64	14	36	39	unknown if m linked (all 4 G1s female)
W5	LVP:1767W5	165	49	169	51	334	57	57	43	43	100	

Supplementary Table 3. Crossing results for TRE-Bn x *AeAct4*-tTAV or *AeCPA*-tTAV.

F0 cross	F1 genotype at L4 larvae stage							
	***AeAct4/CPA*-tTAV;TRE-Bn**		**TRE-Bn**		***AeAct4/CPA*-tTAV**		**WT**	
♀ TRE-BnW5 X ♂ *AeAct4*-tTAV	124		150		114		150	
♀ TRE-BnW5 X ♂ *AeCPA*-tTAV	269		270		274		253	
**F0 cross**	**F1 female genotype and flight ability**							
	***AeAct4*-tTAV;TRE-Bn**		**TRE-Bn**		***AeAct4*-tTAV**		**WT**	
	Flying	Non-flying	Flying	Non-flying	Flying	Non-flying	Flying	Non-flying
♀ TRE-BnW5 X ♂ *AeAct4*-tTAV	0	47	47	2	44	3	44	2

Supplementary Table 4. Fertility and fecundity of AeCPA-tTAV;TRE-Bn females.

F0 cross: ♀ TRE-BnW5 X ♂ AeCPA-tTAV								
**F1 female replicate**	**F1 female genotype, fecundity, and fertility**							
	***AeCPA*-tTAV;TRE-Bn**		**TRE-Bn**		***AeCPA*-tTAV**		**WT**	
	No. of eggs laid	No. of hatched larvae	No. of eggs laid	No. of hatched larvae	No. of eggs laid	No. of hatched larvae	No. of eggs laid	No. of hatched larvae
1	91	0	113	73	48	31	91	77
2	98	0	89	66	91	27	63	60
3	44	0	60	45	88	84	36	26
4	50	0	45	28	88	82	80	63
5	65	0	84	51	61	0	30	13
6	76	2	110	108	75	3	116	107
7	75	26	59	56	0	0	61	51
8	55	19	64	6	104	78	83	81
9	59	0	61	51	85	12	46	27
10	145	10	59	49	53	28	53	46
11	74	0	51	43	67	21	72	67
12	53	0	52	51	101	101	46	46
13	78	0	50	39	39	0	66	42
14	67	0	87	86	104	82	87	66
15	80	1	73	66	0	0	85	70
16	64	0	56	34	81	21	109	102
17	68	14	105	105	87	62	96	83
18	64	0	66	64	63	49	78	53
19	59	0	65	65	48	44	76	57
20	71	0	101	77	63	60	56	33
21	54	20	85	79	141	131	68	21
22	57	10	84	64	85	24	57	53

23	77	0	52	14	64	34	81	0
24	57	0	77	55	103	88	22	9
25	63	0	94	63	44	23	61	55
26	72	0	83	77	60	47	55	54
27	0	0	0	0	57	50	68	21
28	66	0	90	90	69	39	92	87
29	97	0	48	46	89	77	101	91
30	84	0	53	37	78	5	41	30
31	92	0	48	14	64	57	54	45
32	66	5	75	65	78	63	87	9
33	54	14	68	67	107	40	100	7
34	29	0	104	68				
35	88	0	71	32				
36	70	0	53	44				
37	92	0	54	43				
38	69	0	73	60				
39	73	4	57	51				

Supplementary Table 5. PUb-Bs rescues trunPUb-tTAV;TRE-Bn.

F0 cross	F1 genotype			
	***trunPUb*-tTAV; PUb-Bs-TRE-Bn**	***PUb*-Bs-TRE-Bn**	***trunPUb*-tTAV**	**WT**
♀ *PUb*-Bs-TRE-BnW5 X ♂ *trunPUb*-tTAV	85	98	94	71

Supplementary Table 6. PUb-Bs rescue of AeAct4-tTAV;TRE-Bn.

<table>
<tr><th rowspan="2">F0 cross</th><th colspan="8">F1 genotype at L4 larvae stage</th></tr>
<tr><th colspan="2">trunPUb-tTAV;TRE-Bn</th><th colspan="2">TRE-Bn</th><th colspan="2">trunPUb-tTAV</th><th colspan="2">WT</th></tr>
<tr><td>♀ TRE-BnW5 X ♂ AeAct4-tTAV</td><td colspan="2">78</td><td colspan="2">107</td><td colspan="2">94</td><td colspan="2">80</td></tr>
<tr><th rowspan="3">F0 cross</th><th colspan="8">F1 female genotype and flight ability</th></tr>
<tr><th colspan="2">trunPUb-tTAV; PUb-Bs-TRE-Bn</th><th colspan="2">PUb-Bs-TRE-Bn</th><th colspan="2">trunPUb-tTAV</th><th colspan="2">WT</th></tr>
<tr><td>Flying</td><td>Non-flying</td><td>Flying</td><td>Non-flying</td><td>Flying</td><td>Non-flying</td><td>Flying</td><td>Non-flying</td></tr>
<tr><td>♀ PUb-Bs-TRE-BnW5 X ♂ AeAct4-tTAV</td><td>17</td><td>29</td><td>47</td><td>2</td><td>46</td><td>2</td><td>45</td><td>2</td></tr>
</table>

References

1. Ramirez, A. L. (male) Aedes aegypti. https://doi.org/10.6084/m9.figshare.7699778.v1 (2019).
2. Ramirez, A. L. Aedes aegypti mosquito. https://doi.org/10.6084/m9.figshare.7155857.v3 (2019).
