# Supplementary material for: Harnessing the highly adaptable barnase-barstar system for genetic biocontrol of Aedes aegypti
Source: Commun Biol. 2025 Aug 4;8:1154. doi: 10.1038/s42003-025-08588-6 (PMC12322067; doi:10.1038/s42003-025-08588-6)
Supplement: Supplementary file 3 — Description of Additional Supplementary Files [file 42003_2025_8588_MOESM3_ESM.docx]

Description of Additional Supplementary Files

**File name:** Supplementary Data 1

**Description:** The source data behind all graphs in the paper.
